# Evaluation of Controlled Release Urea on the Dynamics of Nitrate, Ammonium, and Its Nitrogen Release in Black Soils of Northeast China

**DOI:** 10.3390/ijerph15010119

**Published:** 2018-01-11

**Authors:** Xin Tong, Xueqin He, Hongwei Duan, Lujia Han, Guangqun Huang

**Affiliations:** Laboratory of Biomass & Bioprocessing Engineering, College of Engineering, China Agricultural University, (East Campus), Beijing 100083, China; tong2012@cau.edu.cn (X.T.); hxq12@cau.edu.cn (X.H.); dhwsg123@cau.edu.cn (H.D.); hanlj@cau.edu.cn (L.H.)

**Keywords:** black soils, controlled release urea, nitrate, ammonium, kinetics, correlation

## Abstract

Controlled release urea (CRU) is considered to enhance crop yields while alleviating negative environmental problems caused by the hazardous gas emissions that are associated with high concentrations of ammonium (NH_4_^+^) and nitrate (NO_3_^−^) in black soils. Short-term effects of sulfur-coated urea (SCU) and polyurethane-coated urea (PCU), compared with conventional urea, on NO_3_^−^ and NH_4_^+^ in black soils were studied through the buried bag experiment conducted in an artificial climate chamber. We also investigated nitrogen (N) release kinetics of CRU and correlations between the cumulative N release rate and concentrations of NO_3_^−^ and NH_4_^+^. CRU can reduce concentrations of NO_3_^−^ and NH_4_^+^, and PCU was more effective in maintaining lower soil NO_3_^−^/NH_4_^+^ ratios than SCU and U. Parabolic equation could describe the kinetics of NO_3_^−^ and NH_4_^+^ treated with PCU. The Elovich equation could describe the kinetics of NO_3_^−^ and NH_4_^+^ treated with SCU. The binary linear regression model was established to predict N release from PCU because of significant correlations between the cumulative N release rate and concentrations of NO_3_^−^ and NH_4_^+^. These results provided a methodology and data support for characterizing and predicting the N release from PCU in black soils.

## 1. Introduction

Mollisols are known in other soil classification systems as “black soils” in China. Generally, there are four main major regions of Mollisols on a world-wide basis. The eastern belt among them is best represented in northeast China [1]. Black soils in northeast China are universally known for their good natural fertility and are good for food production [2,3]. In the next 10–15 years, the northeast region is expected to increase China’s grain production by 50% [4,5]. In addition, their physical and chemical properties are far superior when compared with other soils [3,6]. However, over the past few decades, large amounts of chemical fertilizers have been excessively used for increasing crop yields to meet the growing demand for food [7,8,9,10]. Urea, an important synthetic fertilizer, is widely used all over the world as the main source of plant nutrition [11,12,13]. Although conventional urea increases nitrogen (N) content, its efficiency is low because N is only partially absorbed and utilized by plants [13,14]. The remaining N leaves the soil mainly via nitrification, volatilization, and leaching, which is harmful to the environment and has led to a series of agroecological issues [5,15]. Therefore, reducing fertilizer N loss and increasing its utilization efficiency are significant for sustainable agricultural development in the northeast region [2,6,16].

Controlled release urea (CRU) is a new kind of urea that has less-soluble compounds coated the urea core, which enables nutrient release to ideally synchronize with the needs of crops [13]. CRU is considered to enhance crop yield while minimizing the nutrient losses to the environment, thereby alleviating negative environmental problems caused by the hazardous emissions (NH_3_, N_2_O, etc.) [13,17,18,19,20]. However, there are minimal studies about the effects of CRU on the ratio of NO_3_^−^ and NH_4_^+^ in the soil [21]. Actually, the volatilization of NH_3_ is directly proportional to ammonium (NH_4_^+^) concentration in the soil solution [21]. The formation of N_2_O is associated with high nitrate (NO_3_^−^) concentrations in soil [22], and minimizing the accumulation of soil inorganic N (NH_4_^+^, NO_3_^−^, etc.) is expected to reduce N_2_O emissions [23,24]. Thus, it is considerably important to study the accumulation of NO_3_^−^ and NH_4_^+^ in soil under different fertilization treatments for minimizing fertilizer N loss while maximizing its use efficiency [25,26]. The concentrations of NO_3_^−^ and NH_4_^+^ in soils are regulated by numerous factors, such as soil temperature, pH, soil microbiology, fertilizer form, and moisture [27]. It was reported that high application rate of chemical N fertilizer significantly enhanced the amount of NH_4_^+^-^15^N, NO_3_^−^-^15^N in black soils of northeast China, compared to low N application rate [16]. But studies about the effects of CRU, compared with conventional urea, on the accumulation and kinetics of NO_3_^−^ and NH_4_^+^ in black soils of northeast China have not been reported.

In addition, the application of CRU in northeast China is limited by the lack of release kinetics in black soils because any changes in environmental conditions will make the release rate of CRU unpredictable [28]. For sustainable agricultural development in the northeast region, it is necessary to study the release kinetics of CRU in black soils. Although the release rate of CRU in soil has been commonly determined using the weight loss method [29,30,31,32,33], this method presents difficulty in completely separating the soil particles adsorbed on the surface of urea granules and is time-consuming to operate.

The one-month-long experiment using black soils with three N sources was conducted in an artificial climate chamber for the present study. The aims of this research were to (1) compare short-term effects of CRU and conventional urea on the ratio and kinetics of NO_3_^−^ and NH_4_^+^ and (2) investigate the kinetics of N release from CRU and correlations between the cumulative N release rate and concentrations of NO_3_^−^ and NH_4_^+^ in black soils. We hypothesized that (1) CRU could effectively decrease the accumulation of NO_3_^−^ and NH_4_^+^ and maintain the low NO_3_^−^/NH_4_^+^ ratio in soil, compared to conventional urea, and (2) the N release would differ from CRU with two different coatings and concentrations of NO_3_^−^ and NH_4_^+^ could be selected to predict the cumulative N release rate.

## 2. Materials and Methods

### 2.1. Test Materials

Three kinds of urea were selected in this paper as the test fertilizers and are listed in Table 1.

A vibrating sieve was used to separate fertilizer granules and select only those with diameters of 2~4 mm for the test sample. The tested soil was collected from Baishan City, Jilin Province, China, with a geographical location of East longitude 126°7′ to 128°18′ and North latitude 41°21′ to 42°48′. The basic physical and chemical properties of the soil are listed in Table 2 [34,35,36,37,38,39]. 

Plastic pots with an upper diameter of 11.5 cm, lower diameter of 10 cm, and height of 9 cm were used in the experiment. Three treatments were performed with two parallel repeats for the urea group (U), sulfur-coated urea group (SCU), and polyurethane-coated urea group (PCU). The soil samples were mixed and sieved to <2 mm, and 1 kg of soil was placed into each plastic pot. The nitrogen application levels of all fertilization treatments were set to 500 mg·kg^−1^ soil.

### 2.2. Experimental Design

The buried bag method was employed to determine the N release rate of the coated urea in soil [29,30,31,32,33,40,41]. The three kinds of urea were placed into 5 × 5 cm polypropylene bags with 20 mesh cells, which were sealed and buried to a depth of 5~10 cm in the soil [30,32,41]. The experiment was carried out in an artificial climate chamber at a temperature of (25 ± 2) °C, a humidity of 75%, and in 33% visible light. Samples were collected after 7, 14, 21, 28, and 35 days. After the mesh bags were opened, coated urea granules were removed and rinsed with distilled water until the soil particles attached to the fertilizer were washed out, then absorbent paper was used to dry the fertilizer surface. The N release rates of the PCU and SCU were determined via the weight loss method [29,30,31,32,33]. CRU granules were dried at room temperature at least two weeks to a constant weight. Conventional urea was completely dissolved in the soil at the time of the first sampling, and all nitrogen was released. All soil in the pots was collected and mixed evenly after 2 mm sieve, which was stored at −20 °C for test. After extraction with potassium chloride solution, the concentrations of NO_3_^−^ and NH_4_^+^ accumulation in soil were measured with a flow injection analyzer [42].

### 2.3. Data Analysis Methods

The average value was calculated for each treatment. Graphic drawing was performed using Originpro V.8.5 (OriginLab, Northampton, MA, USA) software. Correlation and regression analysis were performed using SPSS Statistics V.17.0 (SPSS, Chicago, IL, USA) and Matlab R2013a (MathWork Inc., Natick, MA, USA) software. The main evaluation parameters of the models include the Pearson correlation coefficient (r), the standard error (SE). For the binary linear regression model, the collinearity test was conducted using tolerance (T) and variance inflation factor (VIF).

## 3. Results

### 3.1. Dynamics of Nitrate and Ammonium

#### 3.1.1. Variation on the Ratio of Nitrate and Ammonium

**Figure 1:**
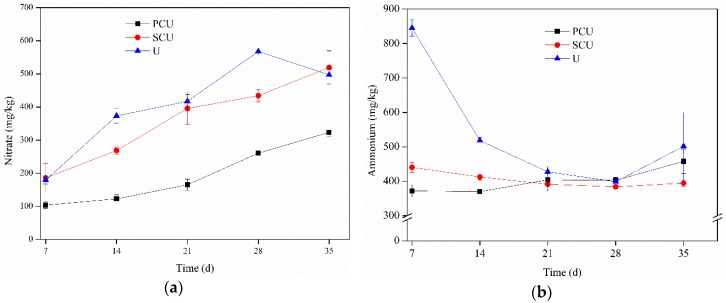
Dynamic changes of (**a**) nitrate and (**b**) ammonium in black soils.

Figure 1 showed concentrations of NO_3_^−^ and NH_4_^+^ accumulation in the soil under three N sources.

As shown in Figure 1a, there was no significant difference between the concentration of NO_3_^−^ with U and SCU treatment in the soil, and both were higher than that of PCU during the experiment. The concentration of NO_3_^−^ in the soil with three fertilization treatments increased gradually before 28 days where the performance of treatments occurred in the following order: U > SCU > PCU. U and PCU treatments decreased after 28 days, and SCU treatment continued to increase until it exceeded U. During the experiment, the accumulation of NO_3_^−^ in the soil treated with PCU was significantly lower than that of U and SCU, which may be due to the fact that N release could be controlled by the polymer coating according to the crop demand. Concentrations of NH_4_^+^ were substantially lower in soils amended with PCU and SCU compared with conventional urea, indicating the slower release of N. The concentration of NH_4_^+^ in the soil with U treatment plummeted in the early stages, followed by a slow drop till 28 days, which was consistent with previous studies. Then, the concentration of NH_4_^+^ with U treatment showed an upward trend that was consistent with previous results. NH_4_^+^ accumulation treated with SCU in black soils gradually declined and then stabilized, while the accumulation of NH_4_^+^ treated with PCU increased slightly, which was consistent with previous results [43].

**Figure 2:**
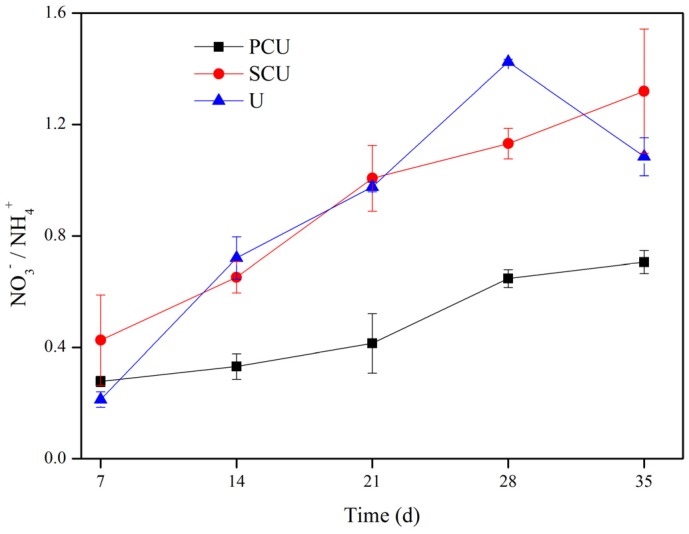
Dynamic changes of the NO_3_^−^/NH_4_^+^ ratio.

Generally, the NO_3_^−^/NH_4_^+^ ratio in soils with three N sources increased during the experiment, and the increasing rate with the application of PCU was lower than with the application of SCU and U (Figure 2). At early stages, the NO_3_^−^/NH_4_^+^ ratios with three treatments were similar, all lower than 1.0. With time, the NO_3_^−^/NH_4_^+^ ratios in the soils treated with U and SCU surpassed 1.0, while the ratio of the soil treated with PCU did not. Obviously, the NO_3_^−^/NH_4_^+^ ratio in soils treated with PCU was lower than that in soils treated with U and SCU, which revealed that PCU was more effective in maintaining lower soil NO_3_^−^/NH_4_^+^ ratios and more suitable for crop growth.

#### 3.1.2. Kinetics of Nitrate and Ammonium

Dynamic parameters related to the NO_3_^-^ and NH_4_^+^ with three N sources were listed in Table 3 and Table 4.

Comparison of the values of Pearson correlation coefficients (r) and the standard error (SE) of the first-order kinetic, Simple Elovich, and Parabolic diffusion equations showed that the NO_3_^−^ accumulation data were better fitted than NH_4_^+^. The high r value and smaller SE of the First-order kinetic equation showed better suitability of this equation for the NO_3_^−^ accumulation in soil treated with U. Similarly, the parabolic diffusion and the simple Elovich equations described well the kinetics of NO_3_^−^ accumulated in soil treated with PCU or SCU. Among three N sources, the parabolic diffusion equation with U treatment has the smallest SE, indicating that the model has the highest accuracy.

The kinetics of NH_4_^+^ accumulation in black soils treated with U could not properly be described by these three kinetic equations because parameters (r = 0.858, SE = 90.49) were not significant. Similar to NO_3_^−^, the parabolic diffusion and the simple Elovich equations described well the kinetics of NH_4_^+^ accumulated in soil treated with PCU or SCU. Although the r values of two equations for fitting NH_4_^+^ were lower compared with equations for fitting NO_3_^−^, the smaller SE reflected the accuracy of equations used to describe the kinetics of NH_4_^+^ was higher.

### 3.2. Dynamics of Nitrogen Release from PCU and SCU

#### 3.2.1. Characterization of Nitrogen Release

**Figure 3:**
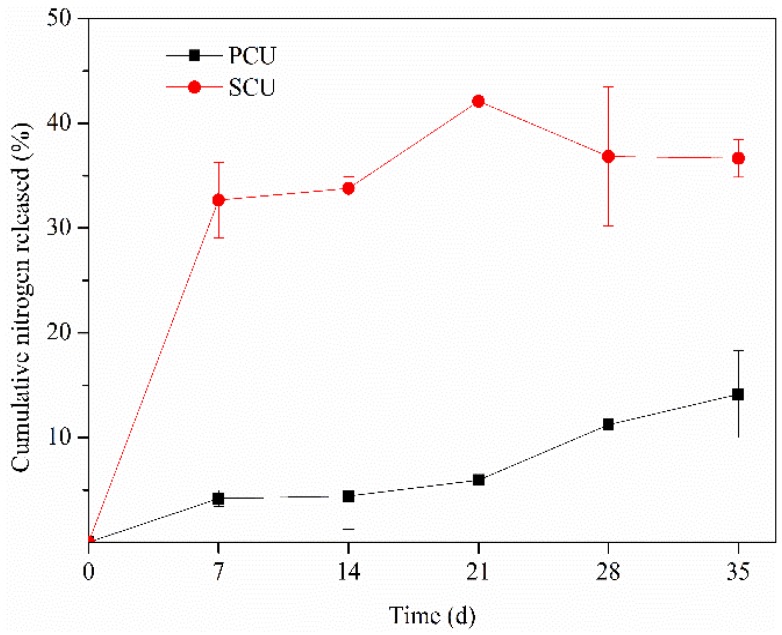
Nitrogen release from polyurethane-coated urea (PCU) and sulfur-coated urea (SCU).

In Figure 3, it is clear that the cumulative N release rate curves of controlled release urea with two different coatings were significantly different.

At the beginning of the experiment, N release of SCU was faster than that of PCU. In the later stage, the cumulative release rate of SCU gradually stabilized, while PCU increased with a significantly higher growth trend than that of SCU. Initially in the experiment, the cumulative N release rate of SCU soared rapidly and was linearly released before the end of seven days. After that, the cumulative N release rate increased more slowly. By contrast, the cumulative N release rate of PCU increased steadily before 21 days, but the N was considered to release faster in later period because of the larger slope of the curve.

#### 3.2.2. Kinetics Analysis

A number of kinetic equations have been used to study nutrients release characteristics of CRU. The parameters related to the frequently used equations are shown in Table 5.

The relationship between the cumulative N release rate and time can be described using first-order kinetic, Elovich, and parabolic diffusion equations. Using the N release data of PCU and SCU (Figure 3), we compared the fitness of these equations. The highest r value (0.946) and smallest SE (1.671) of the first-order kinetic equation among three equations showed best suitability for the release rate of N from the PCU. However, the highest r value and smallest SE among equations for SCU were only 0.634 and 3.272, respectively, which indicated the three equations could not describe the N release well from SCU since the parameters were not remarkable enough.

### 3.3. Correlation Analysis

**Figure 4:**
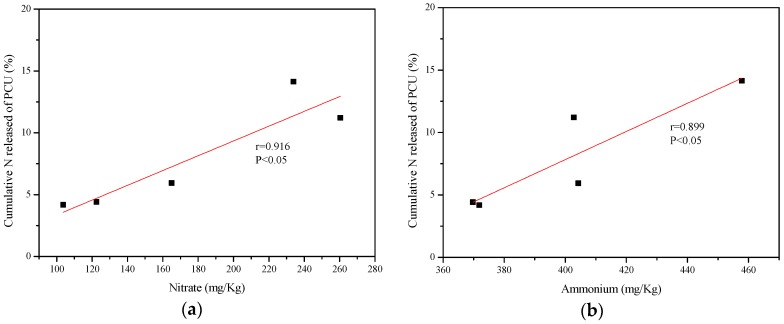
Correlation between cumulative nitrogen release rate of PCU and (**a**) nitrate and (**b**) ammonium in soil.

**Figure 5:**
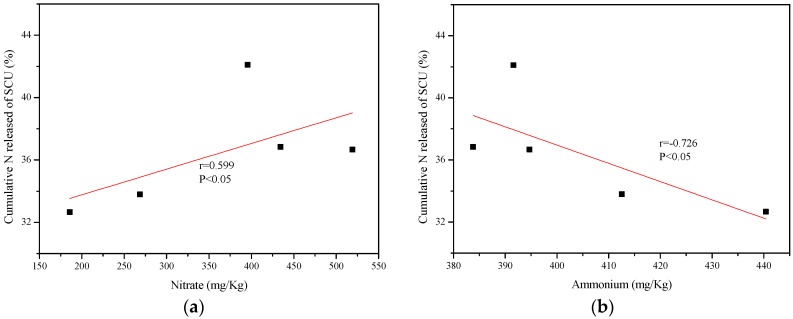
Correlation between cumulative nitrogen release rate of SCU and (**a**) nitrate and (**b**) ammonium in soil.

The correlation analysis between the cumulative N release rate of CRU and accumulation of NO_3_^−^ and NH_4_^+^ in black soils is shown in Figure 4 and Figure 5.

The cumulative N release rate of PCU was positively correlated with concentrations of NO_3_^−^ and NH_4_^+^, and the correlation was significant after bilateral testing at the 0.05 level. Figure 5 showed the N release of SCU and NO_3_^-^ did not have a strong correlation according to the r value, while the N release was negatively correlated with concentrations of NH_4_^+^ after bilateral testing at the 0.05 level.

Based on correlation analysis results, NO_3_^−^ and NH_4_^+^ were selected as the binary variables in the predictive model of N release of PCU and SCU. The models and evaluation results were shown in the Table 6.

The model with good fitness (r = 0.973, SE = 1.449) for PCU reflected the significance of NO_3_^−^ and NH_4_^+^ in determination of cumulative N release rate in black soils, and it did not have collinearity problem. This model can effectively and quantitatively predict the N release of PCU in black soils through with the convenience and accuracy of data acquisition. Unfortunately, the r value (0.731) and SE (3.537) of the model for SCU were not remark enough to further analysis and predict the N release of SCU. Although the model for predicting the N release of PCU had a better fitting degree, the model accuracy need to be improved by increasing the number of samples in further research.

## 4. Discussion

### 4.1. Effects of CRU on Nitrate and Ammonium

Studies have shown that any form of nitrogen from fertilizers that are applied to soil undergoes complex interactions with plant roots, soil microbes, chemical reactions, and loss pathways [21]. It has been reported that NH_4_^+^ is converted to NO_2_^−^ by the oxidation of nitrite bacteria, and NO_2_^−^ is easily oxidized to NO_3_^−^ by nitrified bacteria [44]. NO_3_^−^ in high concentration can be leached or transferred from the zone near plant roots to surface water or groundwater [21,45,46,47], while the remaining NO_3_^−^ under hypoxia conditions is denitrified into N_2_, NO, and N_2_O, which are then dispersed into the atmosphere [17]. It was reported that the concentration of NH_4_^+^ decreased sharply during the incubation period from 10 to 15 days with the application of fertilizer-N [48], which could be due to the loss of NH_3_ by volatilization or transformation of NH_4_^+^ to NO_3_^−^ by nitrification [17,43]. It could be concluded from Figure 1, to some extent, CRU is beneficial in reducing the adverse effects caused by residual N that are not fully utilized after fertilization and transferred through various routes into the environment [13,49].

Generally, the NO_3_^−^/NH_4_^+^ ratio in soils with three N sources increased during the experiment (Figure 2), consistent with the results of previous studies [48], perhaps because NH_4_^+^ was readily converted to NO_3_^−^ by microorganisms [50]. NO_3_^−^ and NH_4_^+^ play an important role in plant growth and seed yield [51]. Lower ratios of NO_3_^−^/NH_4_^+^ was important for uptake of N [50], and could promote vegetative growth rate of plants and floral tiller number [52], as well as increase cereal yields [53]. Fertilizer N source was available to obtain and maintain relatively low soil NO_3_^−^/NH_4_^+^ ratios [50,53,54]. In addition, the ratio of NO_3_^−^/NH_4_^+^ was used as indicators of nitrification inhibition effectiveness [48], a further indication that nitrification was slower with PCU treatment than with SCU and U throughout the study period.

Several equations have been reported to fit and model the release of NO_3_^−^ and NH_4_^+^ with time. Among them, three equations, including the first-order kinetics equation, parabolic diffusion equation, and simple Elovich equation, have been frequently used to study kinetics of NO_3_^−^ and NH_4_^+^ accumulation especially in calcareous soils [55,56]. Parabolic diffusion and simple Elovich equations were reported to describe very well the kinetics of K release from calcareous soils [56]. Because NH_4_^+^ and K^+^ were considered to behave the same in soils [55], these two equations were also selected to study the release kinetics of NH_4_^+^ in calcareous soils and the simple Elovich equation was regarded to fit well the release of NH_4_^+^ [55]. N fertilizer application as one of factors affecting NH_4_^+^ concentration in soil solution may control the release of NH_4_^+^ [55], thus the fate of NH_4_^+^ after the application of conventional urea and CRU has been discussed in this paper.

### 4.2. Characterization and Prediction of Nitrogen Release from CRU in Black Soils

N was released more steadily from PCU than from SCU, indicating that PCU had better controlled release properties of N [57,58,59]. Other studies have shown that the N release profile of PCU in the soil could be inferred from the N released in water at 25 °C [44,60]. It has also been shown that SCU granules release urea in the soil faster than in solution [60]. The release of N in the later stage of the experiment tended to be gentle, which is mainly due to the fact that SCU is controlled by the micropores and cracks in the sulfur coating. Water entered the membrane to dissolve the fertilizer and form a solution, causing the internal pressure of the coated granules to increase. The sulfur shell then ruptured due to its brittleness and inelasticity, leading to rapid release of urea in the short term and slight insufficiency of N supply later in the experiment [60,61]. Although the cumulative N release rates of CRU did not reach 100% at the end of the experiment, the results could be used to characterize the patterns of the N release in black soils from CRU like previous studies [62,63,64,65]. The relationship between the cumulative N release rate and time can be described using first-order kinetic, Elovich, and parabolic diffusion equations [66,67,68]. Using the N release data of PCU and SCU (Figure 3), we compared the fitness of these equations. The highest r value (0.946) and smallest SE (1.671) of the First-order kinetic equation among three equations showed best suitability for the release rate of N from the PCU [69,70]. The first-order kinetic equation was established to predict N release from resin-coated urea in a typical cinnamon soil [71], and it was also used to describe slow-release mechanism of N from organic-inorganic compound–coated urea [67]. However, the fitness of three equations (0.536 < r < 0.634) for SCU was poor mainly because the N release from SCU is controlled by rupture mechanism, while the N release from polymer coating is affected by diffusion [13,28].

Controlled release urea (CRU) was widely reported to increase crop yields while reducing the nitrogen loss and increasing its utilization efficiency [13,17,18,19,20], which is very important for the sustainable development of the environment in northeast China. However, studies about the application of CRF in black soil regions are very limited. Correlation analysis results were conducive to study effects of CRU on reducing environmental pollution in the northeast China. Based on predictive models of N release from PCU, we could study the N release from PCU in black soils by determining the concentrations of NO_3_^−^ and NH_4_^+^ instead of the weight loss method [29,30,31,32,33]. However, we need to improve the accuracy by increasing the number of samples in further research.

## 5. Conclusions

CRU can reduce concentrations of NO_3_^−^ and NH_4_^+^ accumulation in black soils, thereby reducing harmful gas emissions, compared with conventional urea. PCU was more effective in maintaining lower soil NO_3_^−^/NH_4_^+^ ratios, indicating that nitrification was slower with PCU treatment, which meant PCU was more suitable for crop growth than SCU and U. The kinetics of NO_3_^−^ accumulation in black soils treated with U was best described by the first-order kinetics equation, while the three kinetic equations were not suitable for describing the kinetics of NH_4_^+^. Parabolic diffusion equation could fit the kinetics of NO_3_^−^ and NH_4_^+^ accumulation treated with PCU. Simple Elovich equation could fit the kinetics of NO_3_^−^ and NH_4_^+^ accumulation treated with SCU. The relationship between cumulative N release rate of PCU and time could be described best by the first-order kinetics equation, followed by parabolic diffusion equation, and then the simple Elovich equation. However, the kinetics of N release from SCU could not be described well by these three equations. Significant correlations were found between the N release rate of PCU and concentrations of NO_3_^−^ and NH_4_^+^ in black soils, so the binary linear regression model was established to predict N release from PCU. These results were conducive to study effects of CRU on reducing environmental pollution in the northeast China caused by the application of excessive fertilizer, and provided a methodology and data support for characterizing and predicting the N release from PCU in black soils.

## Figures and Tables

**Table 1:** Basic information of tested fertilizers.

Types	Labeled Nitrogen Content (%)	Determined Nitrogen Content (%)	Manufacturer
conventional urea (U)	46.2	46.85	Hanfeng Slow-Release Fertilizer Co., Ltd. Shanghai, China
sulfur coated urea (SCU)	37	39.78	Hanfeng Slow-Release Fertilizer Co., Ltd. Shanghai, China
polyurethane coated urea (PCU)	43	46.04	Audiocodes Technology Co., Ltd. Mianyang, China

**Table 2:** Physical and chemical properties of soil.

Basic Indicators	Values	Unit
Organic matter	538.64	g/kg
Water content	176.47	g/kg
pH	7.34	/
Conductivity (EC)	178.2	us/cm
Bulk density	0.858	g/cm^3^
Porosity	67.62	%
Total nitrogen	6427	mg/kg

**Table 3:** Dynamic characteristics of NO_3_^−^ in soil.

Treatment	Model	Equation	r	SE
U	First-order kinetic	qt = 615.085 (1 − e^−0.06t^)	0.952	46.36
	Simple Elovich	qt = − 33.493 + 220.584 ln(t)	0.950	50.73
	Parabolic diffusion	qt = − 63.984 + 106.174 t^0.5^	0.926	59.58
PCU	First-order kinetic	qt = 332.770 (1 − e^−0.039t^)	0.917	30.79
	Simple Elovich	qt = − 102.950 + 96.473 ln(t)	0.897	31.32
	Parabolic diffusion	qt = − 38.947 + 48.720 t^0.5^	0.918	28.8
SCU	First-order kinetic	qt = 676.778 (1 − e^−0.040t^)	0.939	54.47
	Simple Elovich	qt = − 235.365 + 205.318 ln(t)	0.950	47.22
	Parabolic diffusion	qt = − 93.994 + 102.525 t^0.5^	0.926	57.53

Significant at *p* < 0.05.

**Table 4:** Dynamic characteristics of NH_4_^+^ in soil.

Treatment	Model	Equation	r	SE
U	First-order kinetic	/	/	/
	Simple Elovich	qt = 1234.959 − 239.999 ln(t)	0.858	90.49
	Parabolic diffusion	qt = 1023.124 − 109.340 t^0.5^	0.709	99.33
PCU	First-order kinetic	qt = 413 (1 − e^−0.302t^)	0.537	21.13
	Simple Elovich	qt = 267.512 + 46.085 ln(t)	0.815	19.57
	Parabolic diffusion	qt = 294.295 + 24.128 t^0.5^	0.866	17.95
SCU	First-order kinetic	/	/	/
	Simple Elovich	qt = 500.964 − 33.184 ln(t)	0.932	8.798
	Parabolic diffusion	qt = 474.185 − 15.684 t^0.5^	0.893	10.5

Significant at *p* < 0.05.

**Table 5:** Kinetics equations, correlation coefficients (r), and standard errors (SE) of nitrogen release of PCU and SCU in soil.

Controlled Release Urea	Model	Equation	r	SE
PCU	First-order kinetic	qt = − 15.829 (1 − e^0.018t^)	0.946	1.671
	Simple Elovich	qt = − 20.2 + 6.001 ln(t)	0.854	2.683
	Parabolic diffusion	qt = − 5.98 + 3.146 t^0.5^	0.907	2.171
SCU	First-order kinetic	qt = 37.76 (1 − e^−0.269t^)	0.634	3.272
	Simple Elovich	qt = 26.64 + 3.364 ln(t)	0.584	3.434
	Parabolic diffusion	qt = 29.65 + 1.524 t^0.5^	0.536	3.571

Significant at *p* < 0.05.

**Table 6:** Linear predictive models for predicting the N release of controlled release urea (CRU).

Regression Equation	Collinearity		r	SE
	T	VIF		
PCU = 0.03618N + 0.06146A − 23.1	0.454	2.203	0.973	1.449
SCU = −0.004514N − 0.1409A + 95.06	0.232	4.310	0.731	3.537

Significant at *p* < 0.05. Variance inflation factor (VIF).
